# Nutzung von Routinedaten unterschiedlicher gesetzlicher Krankenkassen: Ein Erfahrungsbericht aus der TIM-HF2-Studie zur Datenerschließung, -validierung und -aufbereitung

**DOI:** 10.1007/s00103-023-03735-y

**Published:** 2023-06-30

**Authors:** Hanna Winkler, Friedrich Koehler, Thomas Reinhold, Stefan N. Willich, Sandra Prescher

**Affiliations:** 1 Institut für Sozialmedizin, Epidemiologie und Gesundheitsökonomie, Charité – Universitätsmedizin Berlin, Luisenstr. 57, 10117 Berlin, Deutschland; 2 CharitéCentrum 11 (CC 11) für Herz‑, Kreislauf- und Gefäßmedizin, Medizinische Klinik mit Schwerpunkt Kardiologie und Angiologie, Arbeitsbereich kardiovaskuläre Telemedizin, Charité – Universitätsmedizin Berlin, Berlin, Deutschland

**Keywords:** Routinedaten, Gesetzliche Krankenversicherung, Gesundheitsökonomische Evaluation, Herzinsuffizienz, Telemedizin, Routine data, Statutory health insurance, Health economic evaluation, Heart failure, Telemedicine

## Abstract

**Hintergrund:**

Im Rahmen der randomisierten, kontrollierten und klinischen Studie „TIM-HF2“ wurde der Nutzen von Telemonitoring bei chronischer Herzinsuffizienz untersucht. Die gesundheitsökonomische Evaluation dieser Intervention basierte auf Routinedaten von gesetzlichen Krankenkassen. Da die Teilnehmenden unabhängig von ihrer Krankenkassenzugehörigkeit rekrutiert wurden, ergab sich eine Vielzahl von potenziellen datenliefernden Krankenkassen. Daraus resultierten sowohl organisatorische als auch methodische Herausforderungen von der Mitwirkung der Datengeber bis hin zur Datenaufbereitung.

**Methode:**

Von der Studienplanung über die Datenakquise bis hin zur Datenprüfung und -aufbereitung wird die konkrete Vorgehensweise in der TIM-HF2-Studie beschrieben. Es werden mögliche Probleme für die Datenvollständigkeit und Datenqualität identifiziert und erfolgte Lösungsansätze dargestellt.

**Ergebnisse:**

Insgesamt waren die Teilnehmenden bei 49 unterschiedlichen gesetzlichen Krankenkassen versichert, die für insgesamt 1450 Teilnehmende Routinedaten lieferten. Etwa die Hälfte aller initialen Datenlieferungen erfolgte korrekt. Die häufigsten Probleme in der Datenaufbereitung traten bei der maschinellen Lesbarkeit der Daten auf. Erfolgsfaktoren für eine hohe Datenvollständigkeit waren die engmaschige Kommunikation mit den datenliefernden Krankenkassen sowie ein hoher zeitlicher und personeller Einsatz für intensive Datenprüfung und -aufbereitung.

**Diskussion:**

Basierend auf den Erfahrungen der TIM-HF2-Studie ist eine große Heterogenität in der Vorhaltung und Übermittlung von Routinedaten erkennbar. Für einen effizienteren Datenzugang, zur Verbesserung der Datenqualität und zur schnelleren Nutzbarkeit für Forschungszwecke wäre eine allgemeingültige Datenbeschreibung wünschenswert.

## Abstract (en)

**Background:**

The randomized controlled clinical trial “TIM-HF2” investigated the benefit of telemonitoring in chronic heart failure. The health economic evaluation of this intervention was based on routine data from statutory health insurance (SHI) funds. Since participants were recruited independently of their SHI affiliation, there was a large number of potential data-providing SHI funds. This resulted in both organizational and methodological challenges, from participation of the data providers to data preparation.

**Method:**

The procedures are described from study planning and data acquisition to data review and processing in the TIM-HF2 trial. Based on the identification of potential problems for data completeness and data quality, possible solutions have been derived.

**Results:**

In total, participants were insured with 49 different SHI funds, which provided routine data for a total of 1450 participants. About half of all initial data deliveries were correct. The most common problems in data preparation occurred in the machine readability of the data. Success factors for a high level of data completeness were close communication with the SHI funds and a high level of time and personnel commitment to intensive data checking and preparation.

**Discussion:**

Based on the experience of the TIM-HF2 trial, a high heterogeneity has been detected in data management and transmission of routine data. Universally applicable data descriptions are desired to improve data access, quality, and usability for research purposes.

## Hintergrund

Die Nutzung von Routinedaten der gesetzlichen Krankenversicherungen (GKV) ist im deutschen Gesundheitswesen seit vielen Jahren eine in der Versorgungsforschung bewährte Methode, um Maßnahmen und Interventionen unter Alltagsbedingungen zu evaluieren. Der technische Fortschritt in der Datenverarbeitung hat nicht nur die Verwendung von Routinedaten der GKV erleichtert, sondern auch die Verknüpfung mit anderen Datenquellen wie Primär- oder Sekundärdaten anderer Dateneigner vereinfacht.

Viele Evaluationsstudien, insbesondere auch im Rahmen deutscher Innovationsfondsprojekte des Gemeinsamen Bundesausschusses, nutzen heute GKV-Routinedaten zumeist von einer einzigen oder einer geringen Zahl von Krankenkassen. Diese geringe Anzahl und die Tatsache, dass die teilnehmenden Krankenkassen im Vorfeld der Studiendurchführung bereits bekannt sind, ermöglicht dezidierte Absprachen zur Datenbereitstellung bereits in der Phase der Studienplanung. Erfahrungen zur Nutzung von Routinedaten mit vielen unterschiedlichen Krankenkassen im Rahmen einer Evaluationsstudie sind bisher nur von March et al. (2020) [1] publiziert worden. In deren Arbeit werden Herausforderungen bei der Datennutzung von 71 Krankenkassen im Rahmen der EVA64-Studie beschrieben, einer sekundärdatenbasierten kontrollierten Kohortenstudie zur Evaluierung von Modellvorhaben zur sektorübergreifenden Versorgung in der Psychiatrie.

Da eine vergleichbare Veröffentlichung zur Verwendung von GKV-Routinedaten im Kontext einer prospektiven randomisiert-kontrollierten Interventionsstudie, bei der die Rekrutierung der Teilnehmenden unabhängig von der Krankenkassenzugehörigkeit stattfand, bisher nicht bekannt ist [1], soll der vorliegende Artikel als Erfahrungsbericht dienen. Die Herausforderungen bei der Datenakquise und -bereitstellung durch die verschiedenen Krankenkassen sowie bei der Datenprüfung, -aufbereitung und -verknüpfung mit den Primärdaten zu einem auswertbaren Gesamtdatensatz sollen beschrieben und diskutiert werden. Grundlage für diesen Artikel sind die Erfahrungen einer gesundheitsökonomischen Analyse im Rahmen der klinischen Studie „TIM-HF2“ (Telemedical Interventional Management in Heart Failure 2).

## Methodik

### Studiendesign

In der randomisiert-kontrollierten, multizentrischen klinischen Studie „TIM-HF2“ (NCT01878630, DRKS00010239) wurde die Wirksamkeit einer telemedizinischen Mitbetreuung von Patientinnen und Patienten mit chronischer Herzinsuffizienz untersucht, die zwischen 2013 und 2018 in teilnehmenden kardiologischen und hausärztlichen Studienzentren bundesweit rekrutiert und ein Jahr nachverfolgt wurden. Die Interessierten wurden sowohl über die Inhalte und den Ablauf der Studie als auch über die Erhebung, Verarbeitung und Auswertung der Primär- und Sekundärdaten aufgeklärt und erhielten eine Studieninformation. Nach Einholung der schriftlichen Einwilligungserklärung fand zu Studieneinschluss eine 1:1-Randomisierung in die Telemedizin-Gruppe oder die Kontrollgruppe statt. Die Haupthypothese der Studie war, dass durch die nicht-invasive telemedizinische Betreuung ungeplante kardiovaskuläre stationäre Aufenthalte vermieden und die Lebenserwartung sowie die Lebensqualität der Teilnehmenden gesteigert werden können. Die Teilnehmenden der Telemedizin-Gruppe erhielten Messgeräte zur täglichen Anwendung. Die erhobenen Studiendaten wurden verschlüsselt und automatisch an das Zentrum für kardiovaskuläre Telemedizin der Charité übermittelt und dort in einer elektronischen Patientenakte gesichert. Die Behandlung von Teilnehmenden der Kontrollgruppe erfolgte alleinig im Rahmen der Routineversorgung gemäß den aktuell gültigen Behandlungsleitlinien. Das komplette Studiendesign ist beschrieben bei Koehler et al. [2]. Basierend auf den klinischen Primärdaten der Studie konnte gezeigt werden, dass der Einsatz der telemedizinischen Mitbetreuung mit einer Reduktion der verlorenen Lebenstage durch ungeplante, kardiovaskuläre Klinikeinweisungen und Tod assoziiert war [3]. Aufbauend auf den Ergebnissen dieser klinischen Studie wurde das Konzept der nicht-invasiven telemedizinischen Mitbetreuung zum 01.01.2022 in den Leistungskatalog der GKV aufgenommen und ist damit Teil der Regelversorgung.

Im Rahmen der Studie wurde eine zusätzliche gesundheitsökonomische Analyse durchgeführt, bei der die Kosten sowie die Kosteneffektivität der zusätzlichen Nutzung der telemedizinischen Versorgung im Vergleich zu alleinigen herkömmlichen Betreuungsansätzen bestimmt werden sollten. Die Versorgungskosten und die Tage, die die Teilnehmenden lebend und außerhalb des Krankenhauses verbrachten, wurden auf Basis der GKV-Routinedaten ermittelt. Zu diesem Zweck wurden die GKV-Routinedaten und die Primärdaten der Studie auf Teilnehmendenebene verknüpft. Die Intervention erwies sich aus GKV-Perspektive gegenüber der alleinigen Standardbehandlung als kosteneffektiv, da ein überlegener klinischer Outcome mit gleichzeitiger Kostenersparnis einherging [4].

### Akquise der datenliefernden Krankenkassen

Da die Teilnehmenden über die hausärztlichen und kardiologischen Studienzentren deutschlandweit und ohne Berücksichtigung ihrer Krankenkassenzugehörigkeit rekrutiert wurden, ergab sich eine Vielzahl an potenziellen Datenlieferanten. Bei der Studienplanung war es somit vorab nicht möglich, mit allen Krankenkassen zielgerichtet die Datensatzbeschreibung für die Lieferung der Routinedaten abzustimmen. Die final zu berücksichtigenden Krankenkassen standen erst mit Abschluss der Rekrutierung fest. Da jedoch die Teilnahme von Versicherten großer Krankenkassen zu erwarten war, wurde im Rahmen der Studienplanung mit 2 als assoziierte Projektpartner beteiligten mitgliederstarken Krankenkassen eine Datensatzbeschreibung für die gesundheitsökonomische Analyse erarbeitet.

Für die Evaluation der klinischen Effektseite wurden die Anzahl und der Zeitraum der Hospitalisierungen zur Bestimmung des primären Endpunktes benötigt. Nach den Erfahrungen aus der Vorgängerstudie TIM-HF-Studie [5] wird ein Drittel der Hospitalisierungen von den Prüfzentren nicht ausreichend in der Studiendokumentation berichtet. Aus diesem Grund wurde in der TIM-HF2-Studie auf die Angabe zu den Krankenhausaufenthalten in den Routinedaten der Krankenkassen zurückgegriffen. Hierzu wurden im Laufe der Studie alle gesetzlichen Krankenkassen, bei denen Studienteilnehmende versichert waren, um Kooperation gebeten. Zum Teil wurde auf Strategien wie Unterstützungsschreiben von politischen Entscheidungsträgern gesetzt, da eine finanzielle Kompensation nicht vorgesehen war. Mit jeder einzelnen Krankenkasse wurde ein Kooperationsvertrag geschlossen. Zum Zweck der rechtskonformen Übermittlung der Routinedaten musste neben der Einwilligung der Patientinnen und Patienten in die Studienteilnahme, die bei Einschluss erfolgte, nachträglich eine zusätzliche Einwilligung zur Übermittlung der Kassendaten der Studienteilnehmenden eingeholt werden.

Zur Nutzung der Kassendaten musste für jede teilnehmende Krankenkasse ein Antrag auf Sozialdatenaustausch nach §75 SGB X bei den zuständigen Aufsichtsbehörden gestellt werden – dies betraf 9 Landesbehörden sowie für bundesmittelbare Krankenkassen das Bundesamt für soziale Sicherung (vormals Bundesversicherungsamt). Soweit möglich wurden die beteiligten Krankenkassen seitens der Studienleitung über den gesamten Prozess von der Antragstellung bis zur Genehmigung unterstützt. Privatversicherte Studienteilnehmende wurden in der gesundheitsökonomischen Analyse nicht berücksichtigt, weil die Versorgungskosten aufgrund der unterschiedlichen Vergütungsstrukturen systematisch von denen der gesetzlich Versicherten abweichen und nur eingeschränkt vergleichbar sind.

### Datenanforderung der GKV-Routinedaten

Nach Abschluss der Studie wurden alle gesetzlichen Krankenkassen, die mindestens einen Studienteilnehmenden versichert hatten, gebeten, die erbrachten Leistungen über den individuellen Studienzeitraum aus den folgenden Versorgungsbereichen zu selektieren: ambulante und stationäre Behandlungen, Arzneimittelverordnungen, Hilfsmittel- und Heilmittelverordnungen, Verordnungen zur häuslichen Krankenpflege, Leistungen zur Rehabilitation nach SGB V, Arbeitsunfähigkeit und Krankengeld sowie Fahrkosten. Eine detaillierte Beschreibung der Variablen dieser einzelnen Leistungsbereiche findet sich unter anderem bei Neubauer et al. [6].

Alle Leistungen, die nicht tagesgenau, sondern nur quartalsgenau dokumentiert werden oder die den Studienzeitraum überlappende Zeiträume betreffen (wie zum Beispiel stationäre Aufenthalte, die während des Studienzeitraums begannen, aber nach Ablauf des Studienzeitraums endeten), sollten mitgeliefert werden, wenn mindestens ein Tag des Leistungszeitraums im Studienzeitraum lag.

Da der Aufwand für die Datenselektion und -übermittlung bei den beteiligten Krankenkassen möglichst gering gehalten werden sollte, beschränkten sich die Vorgaben für die Datenlieferung auf die festgelegten notwendigen Variablen, die damit verbundene Tabellenstruktur sowie die Pseudonymisierung der Daten. Die beteiligten Krankenkassen wurden gebeten die Daten nach Studienende an die Studienleitung zu übermitteln. Die Pseudonymisierung fand über die zu Studienbeginn generierten Studien-IDs statt, um die Verknüpfbarkeit mit den Primärdaten zu gewährleisten.

### Datenprüf- und Aufbereitungsprozesse

Das Vorgehen bei der Datenprüfung und -aufbereitung orientierte sich an den Leitlinien und Empfehlungen „Gute Praxis Sekundärdatenanalyse“ [7]. Nach erfolgter Datenlieferung der einzelnen Krankenkassen wurde zunächst eine Bewertung der Daten basierend auf nachfolgenden Kriterien vorgenommen: maschinelle Lesbarkeit, Einhaltung allgemeiner datenschutzrechtlicher Vorgaben, Verknüpfbarkeit mit Primärdaten, Vollständigkeit der Daten, Verknüpfbarkeit der einzelnen Datentabellen und Plausibilität der Daten.

Der Prozess der Datenprüfung und -aufbereitung erfolgte in 5 Schritten:

1. *Formale Prüfung: *Zunächst wurde geklärt, ob die gelieferten Routinedaten den datenschutzrechtlichen Vorgaben entsprechen, den richtigen Studienteilnehmenden zugeordnet werden konnten und die korrekten individuellen Studienzeiträume abgedeckt waren. Damit in Verbindung stand die Prüfung der korrekten Pseudonymisierung und die Beurteilung der Maschinenlesbarkeit.
2. *Verknüpfbarkeit:* Es wurde überprüft, ob die übermittelten Routinedaten mit den Primärdaten verknüpfbar waren und hinsichtlich der Angaben in den Stammdaten (Alter, Geschlecht) übereinstimmten. Die Verknüpfbarkeit beider Datenquellen war für die geplante Kosteneffektivitätsanalyse essenziell.
3. *Vollständigkeit:* Nun wurde untersucht, ob Daten für alle Leistungsbereiche und für alle Versicherten und für den kompletten Studienzeitraum entsprechend der Datensatzbeschreibung geliefert wurden.
4. *Übertragbarkeit:* Diese Prüfung umfasste die Beurteilung der Übertragbarkeit und die Aufbereitung der Daten in eine einheitliche vorab definierte Datensatzstruktur. Es wurde untersucht, ob bei der Lieferung von Untertabellen für einen Leistungsbereich entsprechende Schlüssel für die Verknüpfung der Tabellen vorlagen. Bei abweichender Variablenanzahl wurde zusätzlich geprüft, ob der Inhalt einer Variable auf mehrere Variablen verteilt wurde oder umgekehrt.
5. *Plausibilität:* Es war zu prüfen, ob die Kodierung der Variablen eindeutig war und ob die gängigen Klassifizierungssysteme (ICD-10, EBM-GOP, DRG) eingehalten wurden. Darüber hinaus wurde die Validität der Leistungszeiträume geprüft und die Kosten als zentraler Parameter hinsichtlich Auffälligkeiten analysiert.

Ziel dieses Vorgehens war es, häufig auftretende Probleme zu identifizieren und auf dieser Grundlage Lösungsansätze für die Datenaufbereitung abzuleiten, die im Spannungsfeld zwischen optimaler Datenqualität und verfügbarem Zeit- und Ressourcenaufwand angemessen erschienen. Diese Lösungsansätze sind anschließend in den Prozess der Datenaufbereitung eingeflossen. Der Prozess der Datenaufbereitung fand in Tranchen statt, da seit Studienende bis zur Erlangung aller Datenlieferungen bis zu 3 Jahre vergingen.

## Ergebnisse

**Figure 1:**
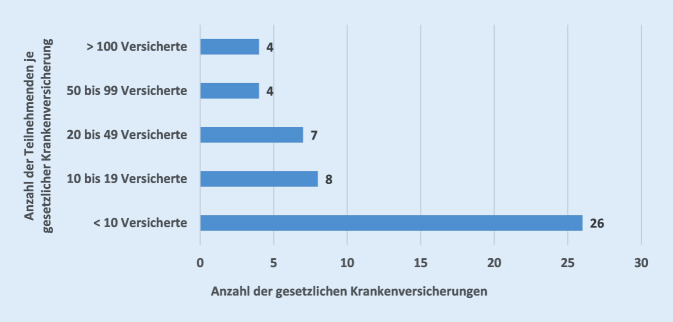

Von den insgesamt 1538 Studienteilnehmenden waren 95 % gesetzlich krankenversichert. Diese verteilten sich auf 49 unterschiedliche gesetzliche Krankenkassen. Insgesamt konnte von allen gesetzlichen Krankenkassen eine Routinedatenlieferung für die bei ihnen versicherten Studienteilnehmenden realisiert werden, sodass finale Datenlieferungen für 1450 Teilnehmende der Studie vorlagen. Die gesetzlich krankenversicherten Studienteilnehmenden verteilten sich – im Sinne der Randomisierung auf Individualebene – nahezu paritätisch auf die beiden Gruppen (*n* = 715 Telemedizin-Gruppe 93,5 %, *n* = 735 Kontrollgruppe 95,1 %). Bezogen auf alle gesetzlichen Versicherten konnten für 99,2 % der Teilnehmenden Routinedaten geliefert werden. Für die fehlenden 11 Teilnehmenden (0,8 %) war aus rechtlichen Gründen (z. B. fehlende Einwilligung der Teilnehmenden zur Datenübermittlung) die Lieferung der Routinedaten nicht möglich. Bei der Verteilung der teilnehmenden Versicherten je Krankenkasse fiel auf, dass mehr als die Hälfte der datenliefernden Krankenkassen weniger als 10 Studienteilnehmende versichert hatten (Abb. 1).

### Erfahrungen aus dem Prozess der Datenakquise

Im Hinblick auf die Datenakquise zeigte sich, dass die einzelnen Krankenkassen zum Teil sehr unterschiedliche rechtliche Anforderungen hatten. So war es im Projekt nicht möglich, eine Standardversion für einen Kooperationsvertrag zu schaffen, der von allen Krankenkassen akzeptiert werden konnte. Deshalb waren mehrere krankenkassenindividuelle Anpassungen notwendig. Eine weitere Herausforderung waren die wechselnden Zuständigkeiten innerhalb der Krankenkassen über den langen Zeitraum der Datenerhebung.

Nur durch den regelmäßigen Kontakt mit allen beteiligten Krankenkassen, der schon während des gesamten Studienzeitraums bezüglich der Akquise der Daten zur Bestimmung der Hospitalisierungen erforderlich war, konnten strukturelle oder personelle Veränderungen bei den Krankenkassen schneller nachvollzogen werden und neue Ansprechpersonen identifiziert werden.

### Erfahrungen aus dem Prozess der Datenprüfung und -aufbereitung

Bei der initialen Datenlieferung erfolgten die Übermittlungen bei 23 der 49 Krankenkassen (46,9 %) vollständig und korrekt gemäß der Datensatzbeschreibung, sodass die Daten ohne weitere Nachforderungen bei der Krankenkasse oder weitere Aufbereitungsarbeiten in die Analysedatenbank übernommen werden konnten.

Die 3 häufigsten Probleme, die sich im Rahmen der Prüfprozesse manifestierten, waren, wie in Tab. 1 dargestellt, (1) die Übermittlung der Daten in nicht-maschinenlesbaren Datenformaten oder ohne vorhandene Datenbankstruktur, (2) das Fehlen von Daten für einzelne Leistungsbereiche und (3) fehlende oder fehlerhafte Kostenvariablen. Insgesamt gab es bei 26 Krankenkassen solche Probleme. Davon gab es bei der Hälfte eines der aufgeführten Probleme, bei der anderen Hälfte 2 oder alle 3 Probleme.

**Table 1:** 

Problembezeichnung	Anzahl und Anteil der betroffenen Krankenkassen (*n* = 49); *n* (%)	Anzahl und Anteil der betroffenen Studienteilnehmenden (*n* = 1450); *n* (%)
**Lieferung in nicht-maschinenlesbarem Datenformat oder ohne Datenbankstruktur**	17 (35)	91 (7)
**Fehlende Datentabellen für einzelne Leistungsbereiche (insgesamt)**	11 (22)	256 (18)
*Nach Leistungsbereich*		
Häusliche Krankenpflege nach SGB V	6	
Rehabilitation nach SGB V	5	
Stationäre Behandlungen	2	
Arbeitsunfähigkeit und Krankengeld	2	
Ambulante Behandlungen	1	
Arzneimittelverordnungen	1	
Fahrkosten	1	
**Fehlerhafte oder fehlende Variablen**	8 (16)	175 (13)

Wenn im Rahmen der formalen Prüfung der Datenlieferung eine mangelnde oder mangelhafte Pseudonymisierung, falsche Datenzeiträume oder -lieferungen für Versicherte auffielen, die sich im Anschluss nicht eindeutig einem Studienteilnehmenden zuordnen ließen, führte dies gemäß der allgemeinen datenschutzrechtlichen Bestimmung zur unmittelbaren Löschung der Daten. Anschließend wurde das festgestellte Problem der betroffenen Krankenkasse mitgeteilt und um eine neue, korrekte Lieferung gebeten.

Daten, die in einem nicht-maschinenlesbaren Datenformat geliefert wurden oder keine Datenbankstruktur aufwiesen, wurden manuell erfasst bzw. formatiert. Dies war ausschließlich bei Datenlieferungen von Krankenkassen mit weniger als 15 versicherten Studienteilnehmenden erforderlich. Die manuelle Erfassung war insbesondere nach Übermittlung von eingescannten Abrechnungsnachweisen oder von Versichertenauskünften notwendig. Bei einer Versichertenauskunft (auch „Patientenquittung“ genannt) handelt es sich um eine Auflistung aller Leistungen, die von der Kasse für den einzelnen Versicherten in einem vordefinierten Zeitraum erstattet wurden. Da dieses Dokument auch auf Nachfrage von den Versicherten angefordert werden kann, gibt es hierfür eine standardisierte Routine bei den Kassen.

Zum Teil wurde um die Lieferung von Versichertenauskünften ausdrücklich gebeten, insbesondere in Fällen, in denen aufgrund manueller Datenselektion (nicht-automatisierte Datenabfrage) Leistungsdaten in späteren Prüfschritten als offensichtlich unvollständig auffielen oder zu stark aggregiert wurden. Hier konnte durch die Erfassung von Versichertenauskünften die Datenqualität deutlich erhöht werden, auch wenn dies mit zusätzlichen zeitlichen und personellen Ressourcen in der Datenaufbereitung einherging.

Wurde bei der Überprüfung der Datenverknüpfbarkeit zwischen Routinedaten und Primärdaten festgestellt, dass Pseudonyme aus den Routinedaten mit den Angaben in den Primärdaten der Studie nicht deckungsgleich waren, wurde zunächst unter Zuhilfenahme von Alter und Geschlecht untersucht, ob es sich lediglich um die Übertragung eines falschen Pseudonyms handelte oder ob bei der Datenselektion ein Fehler aufgetreten war. Je nach Ergebnis des Abgleichs wurde die betreffende Krankenkasse kontaktiert und dort ggf. Daten nachgefordert.

Auch wenn für einzelne Leistungsbereiche keine Daten übermittelt wurden oder einzelne für die spätere Kosteneffektivitätsanalyse unabdingbare Variablen fehlten, wurden die fehlenden Daten bei der betreffenden Krankenkasse nachgefordert. Da eine regelmäßige ambulante Betreuung und eine dauerhafte medikamentöse Behandlung bei chronischer Herzinsuffizienz notwendig sind, wurde beispielweise überprüft, ob für alle Versicherten in der Datenlieferung Leistungsdaten zu ambulanten Behandlungen und Arzneimitteldaten vorlagen. Wenn diese Informationen fehlten, konnte das mit hoher Wahrscheinlichkeit auf Fehler im Selektionsprozess zurückgeführt werden. Bei Hinweisen auf fehlende Datensätze wurde der Kontakt zur Krankenkasse gesucht und bei Bedarf Daten nachgefordert.

Bei der Übertragung aller Daten in eine einheitliche Datenbankstruktur galt das Prinzip des kleinsten gemeinsamen Nenners. Das bedeutet, wenn Angaben einer Krankenkasse vertretbar weniger detailliert vorlagen als bei anderen Krankenkassen, wurde der Detailgrad im Gesamtdatensatz soweit vereinheitlicht bzw. vergröbert, dass die Angaben aller Krankenkassen gemeinsam analysiert werden konnten.

War die Bedeutung der Kodierung von Variablen unklar bzw. unbekannt, wurden verschiedene Validierungsoptionen geprüft. Im Idealfall konnten die Unklarheiten im direkten Kontakt mit der jeweiligen Krankenkasse geklärt werden. Falls nur einzelne bzw. wenige Merkmalsausprägungen in ihrer Kodierung unklar waren, konnte ein Vergleich der Häufigkeitsverteilungen dieser Variable verschiedener anderer Krankenkassen zur Validierung der Kodierung vorgenommen werden. Bei der Prüfung fiel zum Teil auf, dass der Inhalt mehrerer vordefinierter Variablen in einer einzigen der gelieferten Variablen zusammengefasst war (z. B. waren Krankenhaushauptdiagnose und Nebendiagnosen in einer einzigen Diagnose-Variable zusammengefasst) oder dass umgekehrt der Inhalt einer vordefinierten Variable in mehreren Variablen geliefert wurde. In diesen Fällen fand vor der Übertragung in die Analysedatenbank eine Umkodierung der betroffenen zumeist nicht numerischen Variablen (z. B. bei Diagnosecodes, Datumsangaben oder Abrechnungsziffern) statt.

In der Plausibilitätsprüfung fand zunächst eine deskriptive Analyse aller relevanten Variablen statt. Der Fokus bei dieser Prüfung lag insbesondere auf den Kosten in den einzelnen Leistungsbereichen. So wurde bei negativen Beträgen, Nullkosten und Ausreißern geprüft, ob die Beträge unter Berücksichtigung der jeweiligen Leistungsart valide waren. Zum Beispiel wurden bei Auffälligkeiten in den Kosten für den stationären Leistungsbereich die einzelnen Kostenangaben über die DRG(Diagnosis-related Groups)-Erlöse plausibilisiert. Der DRG-Erlös lässt sich für jeden stationären Aufenthalt über die in den Daten vorhandenen Angaben zur jeweiligen DRG bestimmen. Bei systematischen Auffälligkeiten auf Ebene einer einzelnen Krankenkasse wurde diese um Klärung gebeten. Wenn in Einzelfällen keine Validierung unplausibler Angaben vorgenommen werden konnte oder diese zu keinem Ergebnis führte, kam es zum Ausschluss des entsprechenden Falls aus dem Analysedatensatz des betroffenen Leistungsbereiches. Insgesamt konnten 96 % der gelieferten Fälle (1391 von 1450 gesetzlich versicherten Studienteilnehmenden) vollständig in die gesundheitsökonomische Analyse einbezogen werden. Die restlichen 4 % mussten aus Teilbereichen der Analyse ausgeschlossen werden.

## Diskussion

Im Rahmen der TIM-HF2-Studie konnte festgestellt werden, dass der überwiegende Teil der Datenlieferungen von 49 gesetzlichen Krankenkassen von sehr guter Qualität war und der festgelegten Datensatzbeschreibung entsprach. Allerdings war trotz zahlreicher gesetzlicher Vorgaben zur Datennutzung und einer standardisierten Datenübermittlung zwischen Leistungserbringern und Krankenkassen keine Einheitlichkeit bei der Datenhaltung, Selektion und Übermittlung der Daten zu Forschungszwecken erkennbar. Alle Krankenkassen sind verpflichtet im Rahmen des Verfahrens zum Risikostrukturausgleich ihre Routinedaten entsprechend den Vorgaben der Risikostruktur-Ausgleichsverordnung (RSAV) dem Bundesamt für Soziale Sicherung zu liefern. Davon ausgehend könnte angenommen werden, dass dieselben Daten auch für Forschungszwecke standardisiert verfügbar sind. In unserer Studie zeigte sich eine Heterogenität zwischen den einzelnen Krankenkassen hinsichtlich der Qualität der Datenlieferungen. Die häufigsten Probleme betrafen die maschinelle Lesbarkeit, eine fehlende Datenbankstruktur sowie unvollständige Tabellen und Variablen. Unsere Erfahrungen decken sich größtenteils mit den bisher publizierten Erfahrungsberichten zur Validität und Aufbereitung von GKV-Routinedaten [1, 8]. So stellten Hartmann et al. [8] in Bezug auf die Datenqualität von GKV-Routinedaten einen geringen Anteil an unplausibler Information in den Daten fest, der sich zumeist durch eine differenzierte und pragmatische Validierung aufklären ließ. March et al. [1] berichten von kassenspezifischen Unterschieden hinsichtlich der übermittelten Routinedaten, die eine aufwendige und individuelle Prüfung der Daten aller Krankenkassen erforderten – Erfahrungen, die auch im vorliegenden Projekt bestätigt werden konnten.

Dass trotz der Nutzung von GKV-Routinedaten in einer großen multizentrischen Studie mit krankenkassenübergreifender Teilnehmenden-Rekrutierung für fast alle eingeschlossenen Patientinnen und Patienten Routinedaten erhalten und verarbeitet wurden, ist ein Alleinstellungsmerkmal der TIM-HF2-Studie. Daraus ergibt sich für die Ergebnisse der gesundheitsökonomischen Analyse eine hohe Relevanz und Übertragbarkeit auf die Population der gesetzlich versicherten Herzinsuffizienz-Patientinnen und -Patienten in Deutschland.

Von den 110 gesetzlichen Krankenkassen, die in Deutschland zum Zeitpunkt des Studienendes im Jahr 2018 verfügbar waren, beteiligten sich 49 Krankenkassen mit Datenlieferungen an der TIM-HF2-Studie. Die beschriebenen Probleme beziehen sich folglich nur auf diese Krankenkassen und sind nicht zwingend auf die gesamte Kassenlandschaft übertragbar. Da die Rekrutierung der Teilnehmenden unabhängig von ihrer Krankenkassenzugehörigkeit stattfand, sind sowohl kleinere als auch mitgliedstarke sowie regionale als auch überregionale Krankenkassen vertreten.

Einige der identifizierten Probleme hätten im Vorfeld vermutlich schon durch die Lieferung von Testdatensätzen frühzeitig aufgedeckt und vor der Datenlieferung am Studienende behoben werden können. Aufgrund der Vielzahl von Krankenkassen und der Tatsache, dass bis zur Rekrutierung der letzten Teilnehmenden nicht genau bekannt war, welche Kassen final vertreten sind, wurde wegen des hohen Aufwands und der nicht vorhandenen Aufwandsentschädigung darauf verzichtet.

Auch war es z. B. aufgrund langwieriger Vertrags- und Antragsverfahren und auch Datenselektionsverfahren bei den Krankenkassen nicht möglich, die Daten von allen Krankenkassen innerhalb des ersten Jahres nach Studienende zu erhalten. So wurden viele Daten erst im Laufe des zweiten und dritten Jahres nach dem Ende der Studie geliefert. Aus diesem Grund wurden die Daten entsprechend dem Zeitpunkt ihrer Übermittlung in Tranchen validiert und aufbereitet. Aus den Erfahrungen der ersten Datenlieferungen wurde der oben beschriebene Prozess für die Datenaufbereitung entwickelt und im Laufe der Zeit durch neue Erkenntnisse erweitert und iterativ auf bestehende Daten übertragen.

## Fazit

Die im Rahmen der TIM-HF2-Studie vorgesehene GKV-Routinedatenlieferung von 49 datenliefernden Krankenkassen zeigte einige administrative und methodische Herausforderungen, die in zukünftigen ähnlich ausgestalteten Studienvorhaben Beachtung finden sollten. Diese betrafen Abweichungen von der vorgegebenen Datensatzbeschreibung, die einen hohen zeitlichen und personellen Aufwand bei der Datenaufbereitung nach sich ziehen. Die häufigsten Probleme in der Datenaufbereitung waren Lieferungen von Daten in einem nicht-maschinenlesbaren Datenformat oder ohne Datenbankstruktur. Bei auftretenden Problemen waren pragmatische Lösungsansätze notwendig, da die Neulieferung von Daten aus organisatorischen und zeitlichen Gründen oft nicht möglich war.

Aus den Erfahrungen der TIM-HF2-Studie lassen sich für ähnlich gelagerte kassenartenübergreifende Studien folgende Empfehlungen formulieren:

- Es ist empfehlenswert, frühzeitig den Kontakt zu den beteiligten Krankenkassen zu suchen, da die Motivation der Krankenkassen und die entsprechenden Vertragsverhandlungen zeitaufwendig sind.
- Für die Akquise, Erfassung, Aufbereitung, Validierung und Plausibilisierung der Daten, aber auch für Nachfragen und Nachforderungen sind ausreichende zeitliche und personelle Ressourcen einzuplanen, die idealerweise schon in der Studienplanung berücksichtigt werden sollten. Ratsam ist es, hinreichend zeitliche Reserven bei der Festlegung des Enddatums der Datennutzungsfrist einzukalkulieren, da die Beantragung einer nachträglichen Fristverlängerung aufwendig ist.
- Durch das Einholen von Testdatenlieferungen können wertvolle Erfahrungen zu Abweichungen von der definierten Datensatzstruktur und Probleme in der Datenqualität gesammelt werden, um bei Bedarf Anpassungen mit den Krankenkassen zu besprechen und die spätere Aufbereitung der Originaldaten zu beschleunigen. Alternativ kann auch schon eine Testdatenlieferung einzelner unterschiedlicher Krankenkassen hilfreich sein, um bereits vorab mögliche Probleme zu erkennen.
- Eine routinemäßige Anforderung von Code-Books für die gelieferten Daten kann Unklarheiten und Mehrdeutigkeiten in der Kodierung der Variablen verhindern und erspart Ressourcen für die Nachfragen zur Deutung.
- Bei Problemen hinsichtlich der Datenselektion und insbesondere bei unvollständigen Daten kann die Nutzung von Versichertenauskünften zur Erhöhung der Datenqualität in Erwägung gezogen werden. Dies ist jedoch nur bei Krankenkassen hilfreich, die vergleichsweise wenige Studienteilnehmende versichert haben, da die manuelle Extraktion der Daten aus den Versichertenauskünften sehr aufwendig ist.
- Im Sinne der zunehmenden Nutzung von GKV-Routinedaten und im Hinblick auf den Aufbau des Forschungsdatenzentrums Gesundheit, dass die Abrechnungsdaten aller gesetzlichen Krankenkassen in Deutschland für die Forschung erschließen will, wäre eine kassenübergreifende Abstimmung für eine vereinheitlichte Datenbeschreibung anzustreben.
